# Using Fiberless, Wearable fNIRS to Monitor Brain Activity in Real-world Cognitive Tasks

**DOI:** 10.3791/53336

**Published:** 2015-12-02

**Authors:** Paola Pinti, Clarisse Aichelburg, Frida Lind, Sarah Power, Elizabeth Swingler, Arcangelo Merla, Antonia Hamilton, Sam Gilbert, Paul Burgess, Ilias Tachtsidis

**Affiliations:** ^1^Department of Medical Physics and Biomedical Engineering, Malet Place Engineering Building, University College London; ^2^Infrared Imaging Lab, Institute for Advanced Biomedical Technology (ITAB), Department of Neuroscience, Imaging and Clinical Sciences, University of Chieti-Pescara; ^3^Institute of Cognitive Neuroscience, Alexandra House, University College London

**Keywords:** Behavior, Issue 106, Near Infrared Spectroscopy, prospective memory, fNIRS, brain, optical topography, fiberless, wearable, executive functions, imaging, life-based experiment, prefrontal

## Abstract

Functional Near Infrared Spectroscopy (fNIRS) is a neuroimaging technique that uses near-infrared light to monitor brain activity. Based on neurovascular coupling, fNIRS is able to measure the haemoglobin concentration changes secondary to neuronal activity. Compared to other neuroimaging techniques, fNIRS represents a good compromise in terms of spatial and temporal resolution. Moreover, it is portable, lightweight, less sensitive to motion artifacts and does not impose significant physical restraints. It is therefore appropriate to monitor a wide range of cognitive tasks (*e.g.*, auditory, gait analysis, social interaction) and different age populations (*e.g.*, new-borns, adults, elderly people). The recent development of fiberless fNIRS devices has opened the way to new applications in neuroscience research. This represents a unique opportunity to study functional activity during real-world tests, which can be more sensitive and accurate in assessing cognitive function and dysfunction than lab-based tests. This study explored the use of fiberless fNIRS to monitor brain activity during a real-world prospective memory task. This protocol is performed outside the lab and brain haemoglobin concentration changes are continuously measured over the prefrontal cortex while the subject walks around in order to accomplish several different tasks.

**Figure:**
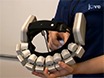


## Introduction

Abnormality of function within prefrontal cortex, and especially the most anterior subpart (rostral prefrontal cortex, or BA10) is common in a range of developmental, psychiatric and neurological conditions. It causes marked disturbances in problem-solving, memory, and attentional abilities in everyday life that are very disabling^1,2^. However, these kinds of problems are difficult to diagnose in the lab or clinic. This is because the mental processes that BA 10 supports are involved in dealing with novel, open-ended situations, where behaviour is self-initiated^3^. Such situations are hard to recreate successfully in the lab, since the formal, artificial and tightly constrained situation the participant typically faces in the lab can change their behaviour and the way that they approach the task. This can significantly reduce the validity of the measurement for either clinical or research purposes, with a strong risk of under-diagnosis^4^. One of the cognitive abilities supported by the frontal lobes where this is most apparent is prospective memory (*i.e.*, the ability to remember to carry out a future action), where it has long been known that there can be significant disagreement between measurements taken in everyday life and the lab^5^. These methodological issues could be largely circumvented if researchers and clinicians investigating prefrontal cortex function, including prospective memory, could do so by taking their measurements in “real-world” situations.

While neuroimaging techniques represent a powerful tool to investigate brain function in a non-invasive and objective way, most of these techniques impose physical constraints on the subject, and are thus not appropriate for use in everyday life settings (*e.g*., functional magnetic resonance (fMRI), magnetoencephalography (MEG), positron emission tomography (PET)). Given the need to bring functional imaging instruments outside the lab and given recent technological improvements, portable and wearable electroencephalography (EEG) and functional near infrared spectroscopy (fNIRS) systems have been developed^6-11^. One of the major advantages of fNIRS over EEG is its higher spatial resolution. Moreover, it is less sensitive to motion artifacts, blinking and eye movements^12^. Wearable fNIRS is thus better suited for use in daily-life contexts, as it imposes fewer physical constraints than EEG and allows free movement in a more natural environment.

fNIRS non-invasively irradiates the head with near-infrared light (650-900 nm). As the biological tissue is relatively transparent in that wavelength range, the light can reach the brain and get absorbed by haemoglobin. fNIRS thus measures the concentration changes of both oxyhemoglobin (HbO_2_) and deoxyhemoglobin (HHb) giving information of oxygenation and haemodynamic changes associated with brain activity. More specifically, brain functional activation is defined as a concurrent increase in HbO_2_ and a decrease in HHb^13^. However, the penetration depth of the light means that signal can only be recovered from the cortical surface. As light is highly diffused in tissue, it is not possible to obtain highly spatially structural information about the brain^14^. Conventional fNIRS systems use optical fibers coupled to the head to guide the light through the scalp and to collect the back-scattered light. Although these instruments are compact, portable and well suited for laboratory settings, optical fibers bundles and their weight limit the movements of the participant and, if not well stabilized, their displacements lead to motion artifact contamination^7^. The new generation of miniaturized and fiberless fNIRS systems offers the possibility to explore brain activity in realistic situations on freely moving participants and without significant physical restraints. Realistic situations are particularly valuable when exploring human executive functions and fiberless fNIRS systems may provide a unique insight into human brain functions. The first fiberless systems were equipped only with a small number of channels (*e.g*., single channel^15 ^and 2 channels^16^) limiting the investigation to small areas. More recently, multichannel wireless and wearable fNIRS devices have been developed^6,7,^^17-20 ^giving the possibility to monitor larger portions of the head on freely moving participants.

In this study, a new multichannel wearable and fiberless fNIRS system was used to monitor and to map prefrontal cortex activity during a real-world prospective memory (PM) task. The fNIRS system is primarily composed of a flexible probe unit (headset) that covers both the dorsolateral and the rostral prefrontal cortex (**Figure 1**), which is connected to a processing unit (portable box) that is worn on the participant’s waist (**Figure 1D**). The headset is made up of 6 surface emitting laser diodes with two wavelength (705 nm and 830 nm) and 6 silicon photodiodes. The absence of optical fibers reduces the weight and the bulk of the probe, being more comfortable and robust against motion artifacts. The optodes are arranged in an alternating geometry (**Figure 1A**) with an inter-optode separation of 3 cm, creating 16 source-detector combinations (*e.g*., 16 measurement channels)^6^. In order to shield the headset from the surrounding light, a shading cap is provided (**Figure 1D**).

The aim of this study was to investigate prefrontal cortex function, during a prospective memory task in the real-world. During prospective memory tasks, participants are asked to remember to respond to an infrequent cue (*e.g*., a familiar face or a parking meter) while performing another demanding task known as an “ongoing task”. In two different blocks of the task, social prospective memory cues (a person) are contrasted to non-social prospective memory cues (a parking meter). This contrast was chosen because it represents a major distinction made between different forms of cue in event-based prospective memory tasks and so the experimental paradigm can be kept close to a “real-life” situation^21^. While BA 10 is known to be sensitive to the processing of social versus non-social information in some situations (*e.g*., Gilbert et al., 2007^22^), recent evidence suggests that haemodynamic changes in BA 10 related to prospective memory tasks are relatively insensitive to cue differences (see Burgess et al., 2011^23^ for review). Thus, it is an open question whether social versus non-social cues affects BA 10 activity in the context of a prospective memory paradigm.

The goal of this study is to evaluate the feasibility of using the fNIRS system to monitor prefrontal cortex hemodynamic and oxygenation changes induced by a real-world cognitive task. Here we report a single case study (one healthy adult participant, 24 years old) on the use of the fNIRS device during a prospective memory task, conducted outside in a typical London street location and mimicking the demands of everyday life. In particular, whether haemodynamic changes in response to social and non-social PM cues can be recorded is investigated.

## Protocol

The protocol was approved by the UCL local research ethics committee, approval number CEHP/2014/901.

### 1. Instruments Setup Prior to the Participant’s Arrival

1. Use video recordings from 3 cameras to analyse “real-world” type tasks (e.g. Shallice and Burgess, 1991^3^):
  1. Place one camera on the experimenter’s chest in order to follow participant’s movements.
  2. Mount the head camera onto the fNIRS shading cap to track where the participant is looking throughout the experiment.
  3. Prepare and turn on the camera for the second experimenter, who follows the first experimenter and the participant for the entire session.
2. Clean the fNIRS headset with a sanitizing wipe.
3. Place a 3D digitizer in an appropriate room (*e.g*., far away from metal objects, walls and floors) and turn it on.

### 2. Participant Preparation and fNIRS Probe Placement

1. Before the experiment starts, have the participant sign the consent form.
2. Use the 10-20 system (**Figure 2**) and digitize the optodes and 10-20 standard positions^24, 25 ^to achieve consistent fNIRS headset placement across all the participants:
  1. Mark with a washable marker the Nasion (Nz, the intersection point between the frontal bone and the nasal bones), Inion (Iz, the occipital protuberance at the back of the scalp) and Left and Right Pre-auricular points (LPA, RPA, the points anterior to the ears in front of the upper end of the tragus) (**Figure 2**) in agreement with manufacturer’s instructions.
  2. Measure the Nz-Iz distance over and around the head and the LPA-RPA distance over the head.
  3. Mark with a washable marker the Cz (the intersection point between the Nz-Iz line and the LPA-RPA line, located at the 50% of the Nz-Iz distance and the 50% of the LPA-RPA distance), Fpz (10% of the Nz-Iz distance) and Fz (30% of the Nz-Iz distance) points based on the 10-20 system (**Figure 2**).
  4. Use a headband with holes matching the optodes positions for a more accurate digitizing across participants. Remove hair from the forehead as much as possible using hair clips along the hairline. Place the digitizing headband over the prefrontal cortex accordingly to the Fpz and Fz points: channel 9 in correspondence of the Fpz point and channel 9-channel 8 line aligned to the Fpz-Fz line (**Figure 1E**).
  5. Digitize the marked 10-20 reference points and the optodes positions by means of the 3D magnetic digitizer.
3. Save the digitized coordinates and use the Spatial Analysis Tool (http://brain-lab.jp/wp/?page_id=52) of the open-source Platform for Optical Topography Analysis Tools (POTATo) software (see the Table of Materials for further information) to register fNIRS data onto a Montreal Neurological Institute (MNI) brain template. **NOTE:** The implemented algorithm for the probabilistic registration converts the digitized locations in the real world coordinate system into the MNI coordinate system and then projects and localizes them onto the MNI brain surface (**Figure 1E**) ^26,27^.
  1. Open POTATo through the Matlab command P3.
  2. Select “Spatial Analysis” from the menu on the main window of the POTATo Graphical User Interface (GUI) and click the “Spatial Analysis” button.
  3. Load the digitized coordinates by clicking the “Empty 10-20” button on the Spatial Analysis Data Viewer window.
  4. Click the “Empty MNI” button.
  5. Select the 10/20 reference points on the MNI estimation window and start the spatial registration.
4. Check for the correct location of the fNIRS channels on the template brain surface (**Figure 1E**): check if channel 8 and channel 9 overlap the inter-hemispheric fissure^28^. If correct, save the channel configuration file for further analyses; otherwise replace the digitizing band re-aligning channels 8 and 9 to the Fpz-Fz line and overlapping channel 9 to Fpz. Then repeat the digitizing procedure.
5. Place the fNIRS headset aligning channels 8 and 9 to the Fpz-Fz line and overlapping channel 9 to Fpz, in agreement with the digitizing headband, and remove the headband (**Figure 1B-C**). Make sure that the probe is well attached to the participant’s head.
6. Place the shading cap with the head camera mounted on it over the fNIRS headset.
7. Explain the experimental rules to the participant. Include device-related precautions (*e.g*., 'Take as little time as possible without rushing or leaving the experimenter behind (NO running)”) as well as task specific rules (*e.g*., 'Do not go outside the Queen Square area into neighbouring streets or areas”).
8. Have the participant successfully memorize all the rules and go outside to start the experiment.

### 3. fNIRS Signals Quality Assessment

1. Use the fNIRS system in wireless mode first to visually inspect signals quality on the fNIRS laptop:
  1. Press the “Power” button on the portable box and turn on the fNIRS in the wireless mode. Open the fNIRS acquisition software on the fNIRS laptop and establish the connection with the portable box.
  2. Press the “Probe Adjustment” button to optimize the detectors gain on the base of the detected light.
  3. Check the probe adjustment results on the software “Probe Adjustment” window and check if each detector receives enough light from the sources by checking if all the channels are classified as “Normal”. If channels are marked as “Stray” or “Under”, re-place the shading cap and maximize the optodes coupling with the forehead. If channels are marked as “Over”, set the power of the laser source to “low”. **NOTE: **As the lateral channels cover the dorsolateral prefrontal cortex, in some cases it may be necessary to move the hair off the forehead to maximize the received light.
  4. Press the “Ready” button and then “Start” to acquire data for a minute and check if heartbeat (haemoglobin oscillations of ~1 Hz) is visible on concentration signals, which ensures a good signal quality.
2. Turn off the portable box in the wireless mode pressing the “Power” button on it. Press the “Power” button in conjunction with the “Mode” button on the portable box to turn on the fNIRS in the stand-alone mode. **NOTE:** The stand-alone mode ensures that the participant can move freely around the experimental area and avoids the necessity to be close to the fNIRS laptop to maintain the wireless connection.

### 4. Data Acquisition

1. Turn on the head camera and the experimenters’ cameras and start filming. Press the “Probe Adjustment” button on the fNIRS portable box to optimize the detectors gain and then press the “Play/Stop” button to start the fNIRS acquisition (sampling frequency=5 Hz).
2. Add a marker to the fNIRS data manually by using the “Mark” button on the fNIRS portable box in conjunction with an audio trigger (*e.g*., a beep). The audio trigger must be clearly recorded on all video cameras. Then start the experiment. **NOTE:** This allows a robust time synchronisation between the different video cameras and the fNIRS recording.

### 5. Experimental Protocol  

1. Include the following conditions and counterbalance the prospective memory ones across the participants:
  1. Use 3 baseline conditions: **NOTE:** This allows to decouple global haemodynamic and oxygenation changes due to walk-related systemic changes versus more localised responses due to brain (neuronal) function.
    1. For the Rest 1 condition, have the participant stand stationary on the street where the test is conducted, and count the number of stimuli on a piece of paper (*e.g*., use a sheet containing Xs and Os printed on it and have the participant count the number of Os on it).
    2. For the Rest 2 condition, have the participant walk a short distance at a normal walking pace, and make no other demands of him.
    3. For the Baseline condition, have the participant walk around the entire street area where the experiment is conducted. **NOTE**: In our case, the experiment took place in Queen Square, London WC1N, U.K.
  2. For the Uncontaminated Ongoing condition, have the participant walk around the experimental area and count the occurrence of certain items (*e.g*., the number of signs affixed to buildings that contain the word “Queen”).
  3. For the Non-social Prospective Memory condition, have the participant carry out the Ongoing task (*e.g*., have the participant count the number of dates and opening hours affixed to buildings), but in addition, if they came within a specified distance of a parking meter, have them go over to it and touch it.
  4. For the Social Prospective Memory condition, have the participant carry out the Ongoing task (*e.g*., have the participant count the number doorbells), but in addition, have him respond to one of the experimenters who acts as a confederate who moves around into pre-specified positions within the experimental area. Have the participant go over to them and give them a “fist bump” greeting.
  5. Use an additional Ongoing condition (Contaminated Ongoing) after the PM conditions (*e.g*., participants has to count the number of unobstructed stairways within the testing area).
  6. Repeat the two Rest conditions described above in opposite order (Rest 2 and then Rest 1). **NOTE: **This allows evaluation of walk-related systemic changes at the end of the experiment.

### 6. Recover Events from the Videos

1. Download the videos from all cameras and save in mpg4 format.
2. Load the videos from all cameras into ELAN (https://tla.mpi.nl/tools/tla-tools/elan/) and synchronise the videos: use Options/Media Synchronisation Mode and align them based on the time point of the audio trigger.
3. In ELAN, use annotations and press the Tier button on the ELAN main window (referring to groups of annotations,* i.e*., one tier for all social PM targets) to mark events in the video stream.
  1. Watch the synchronised video stream and annotate the start and end of each experimental condition, and use tiers for the point at which each PM target is reached. Use separate tiers for social and non-social PM targets.
  2. Complete the video editing for each participant and use the File/Export As/Interlinear Text to export as a text file all the annotated time points.

### 7. Data Analysis

1. Open the fNIRS software and export data from the portable box flash card into the fNIRS laptop. **NOTE:** The fNIRS system processing unit uses the modified Beer-Lambert law and calculates the relative changes in HbO_2_ and HHb from an arbitrary zero baseline at the beginning of the measurement period. Concentration values are hence expressed in molar concentrations (mmol/l) multiplied by the path length (mm)^6^ as they are not corrected for the optical path length.
2. Save concentrations data and import them into Matlab through an in-house pre-processing software.
3. Pre-process the signals following these steps (**Figure 3B**):
  1. Signals down sampling to 1 Hz:
    1. Use a spline interpolation (Matlab function: interp1) to down sample data from 5 Hz to 1 Hz.
  2. Linear Detrending:
    1. To remove slow drifts of the signal, use a linear interpolation (Matlab function: polyfit) between the Rest 1 phases at the beginning and the end of the experiment.
  3. Motion Artifact Correction:
    1. For each channel, identify and remove motion artifacts through a wavelet-based method^31^. Improve signals quality by applying the Correlation Based Signal Improvement (CBSI) method^32^.
  4. Complex wavelet transform:
    1. Use a Morlet mother wavelet, scaled and translated over time, to compute the wavelet transform of each channel through the wavelet toolbox (Matlab function: wt) provided by Grinsted *et al*.^33^ (http://noc.ac.uk/using-science/crosswavelet-wavelet-coherence). **NOTE: **From the wavelet spectrum, it is possible to evaluate the spectral content of signals in a time-frequency space.
  5. Band-pass Filtering:
    1. On the base of the wavelet analysis, use a 3^rd^ order Butterworth band-pass filter (Matlab functions: butter and filter) with cut-off frequencies of 0.008-0.2 Hz^7,^^34^.

## Representative Results

**Figure 3** presents an example of HbO_2_ and HHb un-processed signals (channel 8) recorded during the life-based PM experiment in this case study (**Figure 3A**) and the corresponding signals (**Figure 3C**) after being pre-processed (**Figure 3B**). **Figure 4** shows the wavelet power spectrum of channel 8 HbO_2 _and HHb signals in which the rectangle indicates the frequency range preserved with the band-pass filter. Considering the fact that the participant was walking outside throughout the experiment and moved his head to perform the task, the fNIRS system was robust against motion artifacts and sunlight. In fact, HbO_2_ increments and HHb decrements can be found in correspondence to non-social (**Figure 3D**) and social (**Figure 3E**) prospective memory events. These trends typically denote functional brain activity^13, 35^. In fact, when a brain area is activated, the neurons’ metabolic demand for oxygen increases with consequent increases in regional cerebral blood flow. As most of the oxygen is delivered to cells through haemoglobin, increments in HbO_2_ and decrease in HHb concentrations are observed during functional brain activity^9^. Regions within the prefrontal cortex that exhibit these trends can be assessed by the spatial distribution of HbO_2_ and HHb concentration values mapped over the forehead (**Figure 5**, **Video 1**, **Video 2**). An example of how brain responses to a social PM event are distributed across all the channels is shown in **Figure 5**. **Figure 5A** and **Figure 5B** report respectively the spatial distribution over the forehead of HbO_2_ and HHb to the social PM event (t=2455 s) while **Figure 5C** and **Figure 5D** report respectively the spatial distribution of HbO_2_ and HHb to the non-social PM event (t=1744 s). **Figure 5** shows regional locations (channels) where an increase in HbO_2_ (red, **Figure 5A-C**) and a decrease in HHb (blue, **Figure 5B-D**) are clearly observable, indicative of increase brain function. An example of how prefrontal cortex activity to social PM and non-social PM events and its distribution across the channels change over time is presented in Video 1 and Video 2. In addition, **Figures 6 and 7** show data from all the channels corresponding to the time windows included in **Video 1** and **Video 2**, respectively.

Walk-related haemodynamic and oxygenation changes can be observed in **Figure 3A**. An apparent HHb increases and HbO_2_ decreases occur during walking conditions and these are removed after pre-processing.


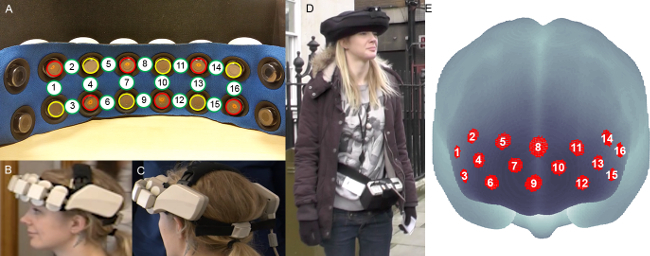
**Figure 1. fNIRS headset placement and channels configuration. **Optodes arrangement in the fNIRS probe is illustrated in panel **A**. Red circles indicate the injection points (sources), yellow circles the collection points (detectors) and green circles the measurements channels. The probe is placed over the forehead (**B, C, D**) with channel 9 in correspondence of the Fpz point and channels 8-9 aligned with the Nasion-Inion midline. The digitized channels location are converted into the MNI coordinate system and overlapped onto the brain cortex (**E**). Please click here to view a larger version of this figure.


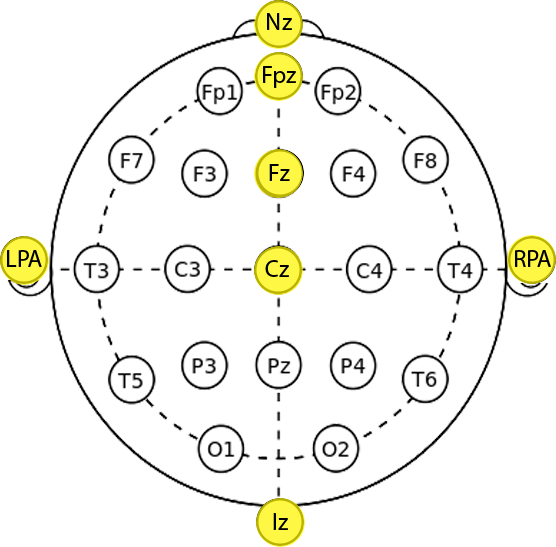
**Figure 2. 10-20 system anatomical references. **Highlighted circles indicate the selected reference points to be marked on the participant’s head (Nz=Nasion, Iz=Inion, LPA=Left Pre-auricular, RPA=Right Pre-auricular). Please click here to view a larger version of this figure.


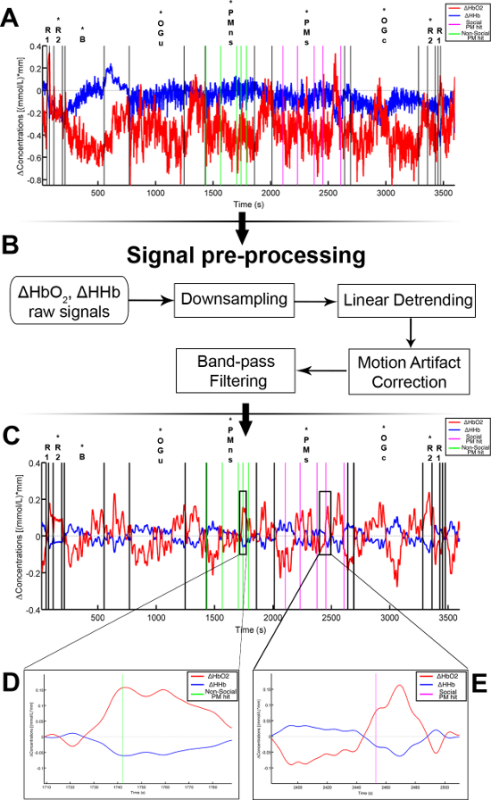
**Figure 3. Signal pre-processing stream. **(**A**) HbO_2_ and HHb raw signals taken from a representative channel (Channel 8).****Black lines mark the start and the end of each experimental condition. Green and magenta lines mark the non-social and social prospective memory hits. Asterisks indicate the walked conditions. (R1=Rest 1; R2=Rest 2; B=Baseline; OGu=Ongoing uncontaminated; PMns=non-social Prospective Memory; PMs=social Prospective Memory; OGc=Ongoing contaminated). (**B**) This panel shows the pre-processing flow-chart applied to Channel 8 raw signals. (**C**) The resulting pre-processed signals are presented. (**D, E**) HbO_2 _increases and HHb decreases occur in response to a chosen non-social (**D**) and social (**E**) prospective memory hits. This hemodynamic trend is usually related to functional activation. Please click here to view a larger version of this figure.


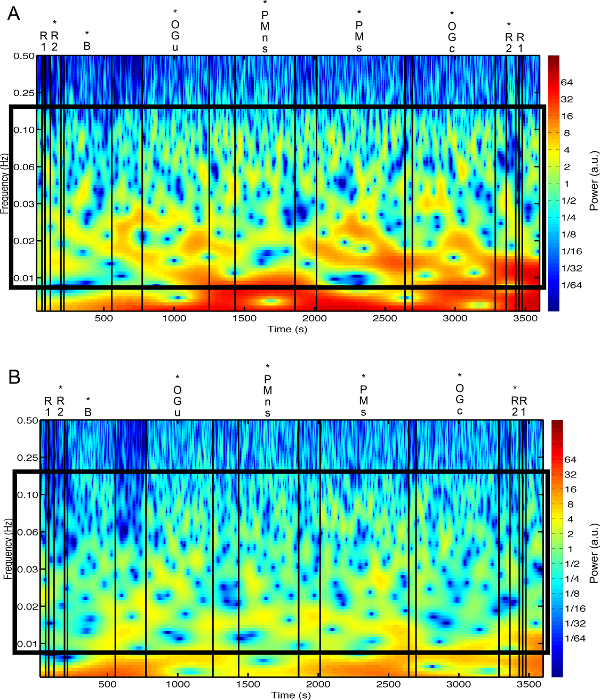
**Figure 4. Wavelet power spectra. **(**A, B**) The wavelet power spectra of channel 8 HbO_2_ and HHb raw signals are presented in panel A and B, respectively.****Black lines mark the start and the end of each experimental condition. Asterisks indicate the walked conditions. (R1=Rest 1; R2=Rest 2; B=Baseline; OGu=Ongoing uncontaminated; PMns=non-social Prospective Memory; PMs=social Prospective Memory; OGc=Ongoing contaminated). The black rectangle highlights the frequency range preserved through the band-pass filter (0.008-0.2 Hz). Please click here to view a larger version of this figure.


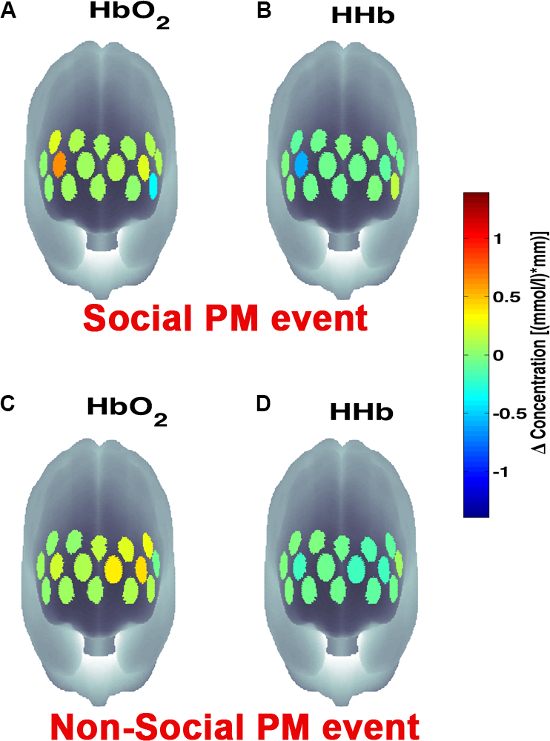
**Figure 5. Spatial distribution of cortical activity to PM events. **HbO_2 _and HHb concentration changes are mapped onto the brain cortex to locate functional activity in response to social PM events (**A-B**) and to non-social PM events (**C-D**). HbO_2 _and HHb values are taken at t=2,455 sec for the social PM event (**A-B**) and t=1,744 sec for the non-social PM event (**C-D**). Please click here to view a larger version of this figure.


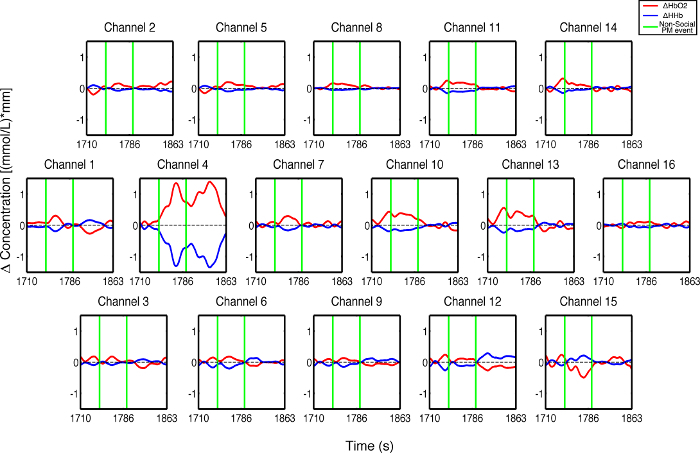
**Figure 6. Oxyhemoglobin and deoxyhemoglobin signals for all the channels in response to non-social PM events. **The green lines indicate the non-social PM events (t=1,744 sec and t=1,792 sec). Please click here to view a larger version of this figure.


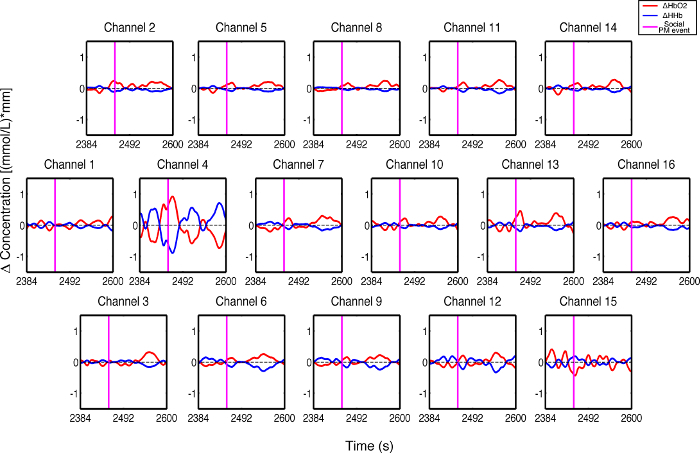
**Figure 7. Oxyhemoglobin and deoxyhemoglobin signals for all the channels in response to a social PM event. **The magenta line indicates the social PM event (t=2,455 sec). Please click here to view a larger version of this figure.


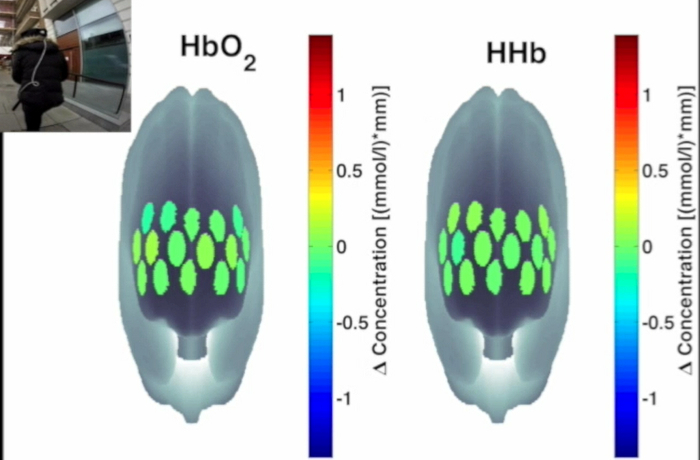
**Video 1. HbO_2_ and HHb concentration changes to social PM events. **The video shows how HbO_2 _(left panel) and HHb (right panel) evolve over time while the participant is approaching to the social PM target. The video of the camera attached to the experimenter’s chest is synchronized.  Please click here to view this video.


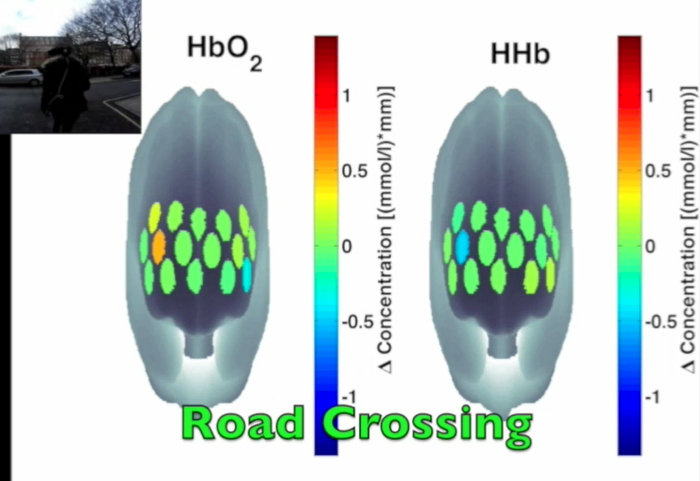
**Video 2. HbO_2_ and HHb concentration changes to non-social PM events. **The video shows how HbO_2 _(left panel) and HHb (right panel) evolve over time while the participant is approaching to the non-social PM target. The video of the camera attached to the experimenter’s chest is synchronized.  Please click here to view this video.

## Discussion

The aim of this study was to evaluate the potential use of wearable and fiberless fNIRS to monitor brain haemodynamic and oxygenation changes related to brain neuronal activity during real-world situations. A wearable and fiberless multichannel fNIRS system was used to measure brain activity over the prefrontal cortex during a prospective memory task performed outside the lab. The case study reported here explored whether brain changes in HbO_2_ and HHb on a freely moving participant in response to social and non-social PM cues in an experiment outside the lab can be monitored continuously and robustly.

The use of fNIRS on freely moving participants in life-based experiments represents a challenging situation. In fact, head movements can cause probe displacements with consequent motion artifacts that corrupt the optical identification of brain activity^36^. Moreover, optical sensors are sensitive to stray light (*e.g*., sunlight when experiments are performed outside), creating additional noise in fNIRS signals. The reported case study provides a preliminary demonstration of the feasibility of the fNIRS system in such real life applications. The absence of optical fibers in such devices prevents optical coupling between the scalp and the optodes resulting in more robust measurements against motion artifacts. In addition, the shading cap ensures a good shielding from the stray light which avoids detectors saturation and low Signal-to-Noise Ratio (SNR). Moreover, increases in HbO_2_ and decrease in HHb concentrations were found in correspondence of social and non-social PM hits (**Figure 3D-E**)^11,^^37^ further supporting its feasibility. In order to assess if the haemodynamic trends observed in **Figure 3D-E** are statistically significant and to locate activated regions within the prefrontal cortex (**Figure 5**, **Video 1**, **Video 2**, **Figure 6**, **Figure 7**), group-level analyses are required. In order to make inference and to identify functionally specialized prefrontal cortex regions^38, 39^, future works will present group data and statistical analyses based on Statistical Parametric Mapping (SPM) using a General Linear Model (GLM) approach.

Even though results have to be considered preliminary, it has been demonstrated that fiberless fNIRS can be effectively brought outside the traditional lab settings and used for real time monitoring of brain activity. This opens up new directions for neurological and neuroscience research. There are at least two obvious areas for application in this respect. The first relates to ecological validity. Cognitive neuroscience researchers investigate patterns of brain activity while people are performing cognitive tasks (using *e.g*., blood oxygen level dependent signal change as a proxy in functional MRI) in order to try to discover how the brain supports our mental abilities. In some cases, it is possible to create experimental situations in the scanner that match very closely the situation in everyday life where the process of interest is used. Consider, for example, reading. Reading words on a display while in a MRI scanner likely makes such similar demands to reading words in a book when at home that it is almost taken for granted that the results gleaned in the scanner can help explain how the brain implements reading in everyday life. However, for many forms of human behaviour and cognition, this assumption is more precarious. For instance, the cognitive processes that a participant uses when a social situation is presented in an MRI scanner (where the participant is immobile, on their own, and in a very unfamiliar and tightly controlled environment) may well be different in important regards to those engaged when the participant is socialising in real life^40^. This is particularly important in social neuroscience where the investigation of the neuronal correlates of inter-personal dynamics (termed *hyperscanning*, for review see Babiloni and Astolfi, 2014^41^) requires a more naturalistic environment. NIRS-based hyperscanning^42, 43 ^may thus represent a new tool to simultaneously monitor brain activity from two or more people in realistic situations. Indeed, there are some mental abilities that cannot be studied well in the highly artificial and physically constrained environment of a MRI, PET or MEG scanner. Those involving ambulation or large amounts of bodily movement as well as those involving social interactions are obvious candidates. For this reason, being able to study the brain activity of participants in naturalistic situations is highly desirable for researchers.

A second, related, broad area of application relates to the use of this technology in clinical situations. An obvious candidate may be neurorehabilitation, where one might wish to study the effects upon the brain of training procedures for activities of daily living (*e.g*., in a kitchen), or of medication upon particular neuronal populations in relation to these activities. But the technology might also perhaps be developed for educational settings as well, and *e.g*., for the use of “real-time” self-monitoring of brain activity. The portability, low risk, and ability to use it in situ in real-world environments with minimal constraint upon behaviour, makes this method very different from others that are currently available.

However, although wearable fNIRS systems show potential for real-world observations, there are other limitations that have to be addressed when using fNIRS during natural walking. Since the infrared light travels through the scalp, it is sensitive to processes that happen both at the cerebral and extra-cerebral compartments of the head. Previous studies demonstrated that a certain amount of the signals measured through fNIRS arises from systemic changes^34, 39, 44 ^that are not directly related to brain activity (see Scholkmann *et al*.^9^ for a review). As intra and extra-cerebral hemodynamic are affected by systemic changes both task-evoked and spontaneous (*e.g*., heart rate, blood pressure, respiration, skin blood flow), physiological changes related to the walking activity should be considered. They originate from the autonomic nervous system (ANS) activity, which regulates heart rate, respiration, blood pressure and vessels diameter through its efferent fibers. More precisely, the sympathetic division of the ANS is hyper-activated during exercise leading to heart rate, blood pressure and respiration increments^45^. For example, previous studies have demonstrated that respiration induces changes in partial pressure of carbon dioxide in the arterial blood (PaCO_2_) which in turn influence cerebral blood flow and cerebral blood volume^46, 47^. In addition, **Figure 3A** shows an example of periodic HHb increases and HbO_2_ decreases that occur within walking periods that can be confused with brain deactivation. In order to make consistent comparisons between conditions (*e.g*., assess if significant changes in concentration occur respect to a baseline period), all the experimental phases should be measured under the same physical activity state. For this reason, a walked rest phase (Rest 2) was included in our life-based protocol. A proper interpretation of fNIRS data requires also a good SNR. This is usually achieved with conventional block and event-related designs where stimulations are repeated several times. Trial repetitions and structured designs are not always possible in life-based experiments. For this reason, additional sensors and appropriate analysis techniques to account for systemic changes^48^ and motion artifacts are necessary to improve the SNR and to correctly interpret brain signals. We plan to investigate the impact of such walk-related systemic changes through the use of portable devices to monitor breathing rate, heart rate and walking pace. Moreover, the problem of events recovery needs to be addressed, too. In cognitive neuroscience experiments, brain activity is investigated in relation to stimuli or environments encountered by participants’, and their behaviour in response to, or anticipation of them. Experimenters therefore need to (a) know what is currently available to the participant in their environment, and (b) have a moment-by-moment record of the participant’s behaviour. In a typical lab situation these factors can be readily controlled since the experimenter can constrain what participants encounter, and the form and number of behaviours that the participant can evince. However, this is not the case in “real-world” environments outside the lab, where many events and experiences that the research participant will have are beyond the strict control of the experimenter^49^. Accordingly, in “real-world” type tasks of the kind studied here, video records are used for analysis (*e.g*., Shallice and Burgess, 1991^3^). This allows to recover both sustained (*e.g*., block-level) and transient (*e.g*., event-related) processes that support different aspects of performance (for review see Gonen-Yaacovi and Burgess, 2012^21^). The events to be recovered from the video recordings will depend on the theoretical question being addressed in the experiment. In the reported case study, event onsets were recovered from the videos filmed by the 3 cameras. This procedure of determining the onset and termination of particular cues and behavioural responses is laborious and requires skill when carried out on life-based data. A central issue is that with “real life” type experiments there is usually not the same degree of a priori knowledge of events as with the lab-based ones, and participants usually have more scope in the way they can respond. Moreover, as participants are free to move in a natural and uncontrolled environment, they are faced with a variety of rapidly-changing stimuli and it is difficult to recover the haemodynamic response to the real event of interest. For example, in the case study, the haemodynamic trends observed for HbO_2_ and HHb (**Figure 3D-E**) are not phase-locked to the video-recovered onset like the typical event-related haemodynamic response^38^. HbO_2_ and HHb start respectively to rise and decrease 20 sec before the stimulus onset and reach a peak after it. Further analyses are thus needed to establish whether PM cues events are happening actually when the participant sees the target, when he approaches towards it or when he reaches it. Given the potential of fiberless fNIRS technologies for real life clinical applications, future work will address the video-coding problem by developing new algorithms to identify event onsets in a more objective way, as well as exploring the possibility of doing it directly from fNIRS data.

## Disclosures

The authors declare that they have no competing financial interests.

## References

[B0] Alvarez JA, Emory E (2006). Executive function and the frontal lobes: a meta-analytic review. Neuropsychol. Rev.

[B1] Jurado MB, Rosselli M (2007). The elusive nature of executive functions: a review of our current understanding. Neuropsychol. Rev.

[B2] Shallice TIM, Burgess PW (1991). Deficits in strategy application following frontal lobe damage in man. Brain.

[B3] Burgess PW, Alderman N, Volle E, Benoit RG, Gilbert SJ (2009). Mesulam's frontal lobe mystery re-examined. Restor. Neurol. Neurosci.

[B4] Kvavilashvili L, Ellis JA (2004). Ecological validity and the real-life/laboratory controversy in memory research: a critical and historical review. History and Philosophy of Psychology.

[B5] Atsumori H (2009). Development of wearable optical topography system for mapping the prefrontal cortex activation. Rev. Sci. Instrum.

[B6] Piper SK (2014). A wearable multi-channel fNIRS system for brain imaging in freely moving subjects. Neuroimage.

[B7] Casson AJ, Smith S, Duncan JS, Rodriguez-Villegas E (2008). Wearable EEG: what is it, why is it needed and what does it entail?. IEEE Eng. Med. Biol. Mag.

[B8] Scholkmann F (2014). A review on continuous wave functional near-infrared spectroscopy and imaging instrumentation and methodology. Neuroimage.

[B9] Hoshi Y (2003). Functional near‐infrared optical imaging: Utility and limitations in human brain mapping. Psychophysiology.

[B10] McKendrick R, Parasuraman R, Ayaz H (2015). Wearable functional near infrared spectroscopy (fNIRS) and transcranial direct current stimulation (tDCS): expanding vistas for neurocognitive augmentation. Front. Syst. Neurosci.

[B11] Lloyd-Fox S, Blasi A, Elwell CE (2010). Illuminating the developing brain: the past, present and future of functional near infrared spectroscopy. Neurosci. Biobehav. Rev.

[B12] Obrig H (2000). Near-infrared spectroscopy: does it function in functional activation studies of the adult brain?. Int. J. Psychophysiol.

[B13] Ferrari M, Quaresima V (2012). A brief review on the history of human functional near-infrared spectroscopy (fNIRS) development and fields of application. Neuroimage.

[B14] Sagara K, Kido K, Ozawa K (2009). Portable single-channel NIRS-based BMI system for motor disabilities' communication tools. Conf. Proc. IEEE Eng. Med. Biol. Soc.

[B15] Shiga T, Yamamoto K, Tanabe K, Nakase Y, Chance B (1997). Study of an algorithm based on model experiments and diffusion theory for a portable tissue oximeter. J. Biomed. Opt.

[B16] Muehlemann T, Haensse D, Wolf M (2008). Wireless miniaturized in-vivo near infrared imaging. Opt. Express.

[B17] Kim CK, Lee S, Koh D, Kim BM (2011). Development of wireless NIRS system with dynamic removal of motion artifacts. Biomed. Eng. Lett.

[B18] Ayaz H, Onaral B, Izzetoglu K, Shewokis PA, McKendrick R, Parasuraman R (2013). Continuous monitoring of brain dynamics with functional near infrared spectroscopy as a tool for neuroergonomic research: empirical examples and a technological development. Front. Hum. Neurosci.

[B19] Safaie J, Grebe R, Moghaddam HA, Wallois F (2013). Toward a fully integrated wireless wearable EEG-NIRS bimodal acquisition system. J. Neural. Eng.

[B20] Gonen-Yaacovi G, Burgess PW (2012). Prospective memory: the future for future intentions. Psychol. Belg.

[B21] Gilbert SJ, Williamson IDM, Dumontheil I, Simons JS, Frith CD, Burgess PW (2007). Distinct regions of medial rostral prefrontal cortex supporting social and nonsocial functions. Soc. Cogn. Affect. Neurosci.

[B22] Burgess PW, Gonen-Yaacovi G, Volle E (2011). Functional neuroimaging studies of prospective memory: What have we learnt so far?. Neuropsychologia.

[B23] Okamoto M (2004). Three-dimensional probabilistic anatomical cranio-cerebral correlation via the international 10–20 system oriented for transcranial functional brain mapping. Neuroimage.

[B24] Jasper HH (1958). The ten twenty electrode system of the international federation. Electroencephalogr. Clin. Neurophysiol.

[B25] Okamoto M, Ippeita D (2005). Automated cortical projection of head-surface locations for transcranial functional brain mapping. Neuroimage.

[B26] Singh AK (2005). Spatial registration of multichannel multi-subject fNIRS data to MNI space without MRI. Neuroimage.

[B27] Koessler L (2009). Automated cortical projection of EEG sensors: anatomical correlation via the international 10–10 system. Neuroimage.

[B28] Burgess PW, Quayle A, Frith CD (2001). Brain regions involved in prospective memory as determined by positron emission tomography. Neuropsychologia.

[B29] Burgess PW, Scott SK, Frith CD (2003). The role of the rostral frontal cortex (area 10) in prospective memory: a lateral versus medial dissociation. Neuropsychologia.

[B30] Molavi B, Dumont GA (2012). Wavelet-based motion artifact removal for functional near-infrared spectroscopy. Physiol. Meas.

[B31] Cui X, Bray S, Reiss AL (2010). Functional near infrared spectroscopy (NIRS) signal improvement based on negative correlation between oxygenated and deoxygenated hemoglobin dynamics. Neuroimage.

[B32] Grinsted A, Moore JC, Jevrejeva S (2004). Application of the cross wavelet transform and wavelet coherence to geophysical time series. Nonlin. Processes Geophys.

[B33] Kirilina E (2013). Identifying and quantifying main components of physiological noise in functional near infrared spectroscopy on the prefrontal cortex. Front. Hum. Neurosci.

[B34] Hoshi Y, Tamura M (1993). Dynamic multichannel near-infrared optical imaging of human brain activity. J. Appl. Physiol.

[B35] Brigadoi S (2014). Motion artifacts in functional near-infrared spectroscopy: a comparison of motion correction techniques applied to real cognitive data. Neuroimage.

[B36] Huppert TJ, Hoge RD, Diamond SG, Franceschini MA, Boas DA (2006). A temporal comparison of BOLD, ASL, and NIRS hemodynamic responses to motor stimuli in adult humans. Neuroimage.

[B37] Friston KJ, Holmes AP, Worsley KJ, Poline JP, Frith CD, Frackowiak RS (1994). Statistical parametric maps in functional imaging: a general linear approach. Hum. Brain Mapp.

[B38] Tachtsidis I, Koh PH, Stubbs C, Elwell CE (2010). Functional optical topography analysis using statistical parametric mapping (SPM) methodology with and without physiological confounds. Adv. Exp. Med. Biol.

[B39] Burgess PW, Alderman N, Evans J, Emslie H, Wilson BA (1998). The ecological validity of tests of executive function. J. Int. Neuropsychol. Soc.

[B40] Babiloni F, Astolfi L (2014). Social neuroscience and hyperscanning techniques: past, present and future. Neurosci. Biobehav. Rev.

[B41] Scholkmann F, Holper L, Wolf U, Wolf M (2013). A new methodical approach in neuroscience: assessing inter-personal brain coupling using functional near-infrared imaging (fNIRI) hyperscanning. Front. Hum. Neurosci.

[B42] Cui X, Bryant DM, Reiss AL (2012). NIRS-based hyperscanning reveals increased interpersonal coherence in superior frontal cortex during cooperation. Neuroimage.

[B43] Tachtsidis I, Leung TS, Devoto L, Delpy DT, Elwell CE (2008). Measurement of frontal lobe functional activation and related systemic effects: a near-infrared spectroscopy investigation. Adv. Exp. Med. Biol.

[B44] Freeman JV, Dewey FE, Hadley DM, Myers J, Froelicher VF (2006). Autonomic nervous system interaction with the cardiovascular system during exercise. Prog. Cardiovasc Dis.

[B45] Scholkmann F, Gerber U, Wolf M, Wolf U (2013). End-tidal CO2: an important parameter for a correct interpretation in functional brain studies using speech tasks. Neuroimage.

[B46] Tisdall MM (2009). The effect on cerebral tissue oxygenation index of changes in the concentrations of inspired oxygen and end-tidal carbon dioxide in healthy adult volunteers. Anesth. Analg.

[B47] Tachtsidis I, Leung TS, Chopra A, Koh PH, Reid CB, Elwell CE (2009). False positives in functional nearinfrared topography. Adv. Exp. Med. Biol.

[B48] Gilbert SJ, Zamenopoulos T, Alexiou K, Johnson JH (2010). Involvement of right dorsolateral prefrontal cortex in ill-structured design cognition: An fMRI study. Brain Res.

